# Preeclampsia induced by cadmium in rats is related to abnormal local glucocorticoid synthesis in placenta

**DOI:** 10.1186/1477-7827-12-77

**Published:** 2014-08-09

**Authors:** Fan Wang, Qiong Zhang, Xiaojie Zhang, Shunqun Luo, Duyun Ye, Yi Guo, Sisi Chen, Yinping Huang

**Affiliations:** Department of Gynaecology and Obstetrics, The Second Affiliated Hospital of Wenzhou Medical University, Wenzhou, 325000 Zhejiang China; Department of Gynaecology and Obstetrics, The First Affiliated Hospital of Wenzhou Medical University, Wenzhou, 325000 Zhejiang China; Department of Biochemistry and Molecular Biology, Tongji Medical College, Huazhong University of Science and Technology, Wuhan, 430030 Hubei China; Department of Pathophysiology, Tonji Medical College, Huazhong University of Science and Technology, Wuhan, 430030 Hubei China

**Keywords:** Cadmium, Preeclampsia, Glucocorticoid synthesis, Placenta, Rat

## Abstract

**Background:**

Cadmium (Cd) is a major environmental pollutant that causes multiple adverse health effects in humans and animals. In this study, we investigated Cd-mediated toxic effects in rats during pregnancy and endocrine intervention in the placenta.

**Methods:**

We exposed pregnant rats to intraperitoneal Cd (CdCl2) at various doses (0, 0.25, and 0.5 mg/kg BW/day) from days 5 to 19 of pregnancy and evaluated the maternal-placental-fetal parameters linked to preeclampsia. We measured the corticosterone level in rat serum and placental tissue by sensitive ELISA and also analyzed the expression of glucocorticoid synthesis enzymes in the placenta.

**Results:**

Key features of preeclampsia (PE), including hypertension, proteinuria, glomerular endotheliosis, placental abnormalities and small fetal size, appeared in pregnant rats after injection with 0.5 mg/kg BW/day Cd. The placental corticosterone production and maternal and fetal plasma corticosterone levels were increased in rats treated with 0.5 mg/kg BW/day Cd (P <0.01). The expression of 21-hydroxylase (*CYP21*) and 11beta-hydroxylase (*CYP11B1)*, enzymes essential for corticosteroid synthesis, were increased in Cd-exposed placenta (P <0.01). 11beta-hydroxysteroid dehydrogenase (*11beta-HSD2*), a dominant negative regulator of local glucocorticoid levels, was decreased in Cd-exposed placenta (P <0.01).

**Conclusions:**

Our study demonstrates for the first time that changes in placental glucocorticoid synthesis induced by Cd exposure during pregnancy could contribute to preeclamptic conditions in rats.

## Background

With increased industrial development, Cd exposure is an increasingly important environmental hazard to both humans and animals [1, 2]. The general population is exposed to Cd daily via the contamination of drinking water and food and by the inhalation of cigarette smoke [3]. Due to its long biological life and low excretion rate, Cd can accumulate in various organs, mainly in the blood, liver, kidney and reproductive systems. Pregnant women are more vulnerable to Cd toxicity due to the greatly increased absorption and retention of Cd caused by nutritional deficiencies in protein and essential minerals (particularly iron, calcium, zinc) during pregnancy [4]. Cd has been implicated in the etiology of essential hypertension, but the relationship between Cd and preeclamptic toxemia remains unclear.

Preeclampsia is a multisystem disorder characterized by hypertension, proteinuria, edema and often fetal growth restriction(FGR) [5, 6]. The syndrome resolves after delivery, suggesting that the placenta is the principal contributor to the pathogenesis of preeclampsia [7]. The placenta is also a primary target for Cd during pregnancy [8]. Cd can accumulate in high concentrations in the placenta with limited transfer to the fetus, although effects on fetal birth size have been observed [9, 10]. The placenta is a temporarily important endocrine organ, producing many hormones that affect the status of the pregnancy and maternal physiology. Cd, similar to some other environmental pollutants, can act as an endocrine disruptor [11]. Several studies have reported that Cd directly affects steroid synthesis in reproductive organs such as the ovary and testes [12, 13]. However, few studies have been conducted to determine the endocrine effect of Cd exposure during pregnancy [14]. Cd exposure during pregnancy increases the levels of circulating corticosterone – the main active glucocorticoids (GC) in rodents – in mothers and offspring, suggesting an involvement of the GC system in some of the toxic effects induced by Cd [15]. Glucocorticoids are steroid hormones and are classically thought to be secreted exclusively by the adrenal glands. However, recent evidence has shown that local corticosteroid synthesis may be triggered in various other tissues during pathological conditions and is therefore regulated by local processes in an adrenal-independent manner [16]. High cortisol levels are associated with hypertension and endothelial dysfunction, features often observed in patients with PE [17]. This paper presents a hypothesis suggesting the involvement of Cd in the etiopathogenesis of preeclampsia based on the endocrine intervention to placenta.

## Methods

### Experimental animals

All animal experiments were approved by the Institutional Animal Care and Use Committee, Tongji Medical College, Wuhan, China. The ethical approval date was December 2, 2012 and the reference number was IACUC [2012]340.

Female Sprague–Dawley rats (10 to 12-weeks old, 200-240 g) were purchased from the Experimental Animal Center of Tongji Medical College, Huazhong University of Science and Technology. Animals were housed individually in plastic cages with wood chips as bedding under pathogen-free conditions in a controlled environment at 20–25°C and 12 hour light/dark cycles. Rats were fed a standard laboratory diet and water ad libitum. The rats were mated overnight with fertile male rats. The next day was taken as day 1 of pregnancy if spermatozoa were found in vaginal smears.

### Maternal and fetal experiments

#### Experimental protocol

Pregnant rats were randomly divided into three groups: the control group (n = 6), the 0.25 mg/kg Cd group (n = 7) and the 0.5 mg/kg Cd group (n = 8). Cd (Sinopharm Chemical Reagent Co. Ltd.) dissolved in sterile saline was administered to the dams in each Cd group by intraperitoneal injection from GD 5 to GD 19. Maternal weight was monitored throughout the dosing period, and the dosing volume was 2 ml/kg body weight. The control group received only sterile saline. Six non-pregnant rats received an intraperitoneal injection of 0.5 mg/kg Cd as non-pregnant controls.

#### Measurement of systolic blood pressure

Systolic blood pressures (SBP) were measured in conscious, restrained pregnant rats in the morning. An automated system with a photoelectric sensor linked to a dual channel recorder (BP-98A, Softron, Japan), tail cuff and sphygmomanometer was used to obtain indirect blood pressure measurements. The measurements were obtained at 4 different time points: before pregnancy and when the animals were 1–4 days, 10–13 days, 19 days pregnant, respectively. For the non-pregnant animals, comparable time periods were utilized.

#### Determination of urinary albumin excretion

On days 3 and 19 of pregnancy, the rats were placed in metabolic cages for 24-hour urine collection. To avoid contaminating the collected urine, rats were restricted from food; however, they were allowed free access to water. To avoid adverse effects of fasting, rats were fed in other cages for 30 min every 6 hours. Urine protein concentrations were determined with a BCA protein assay kit using bovine serum albumin as standard.

#### Specimen collection

On day 20 of pregnancy, rats were anaesthetized between 9:00 a.m. and 10:00 a.m. to avoid hormonal circadian interference. Maternal blood was collected by cardiac puncture and put in polyethylene tubes pre-rinsed with EDTA. Plasma was prepared by centrifugation for 10 min at 3000 g at 4°C. After the rats were killed, pups were delivered by caesarean section. The plasma samples and the remaining placentas were stored at −80°C until analysis.

### Determination of cadmium concentrations

Cd in maternal and fetal blood and placenta tissue was measured by inductively coupled plasma mass spectrometry ICP-MS (ICP-MS; Agilent 7500 with a Cd program) with a detection limit of 15 μg/L for blood and 0.03 μg/g for solid samples.

### Histology and immunohistochemistry

Small parts of the kidneys and placentas harvested from each pregnant rat were randomly selected and fixed horizontally in 10% neutral-buffered formaldehyde solution. After dehydration, the samples were embedded in paraffin, and 4-μm sections were cut by a microtome and collected for routine H&E or immunohistochemical examination. Sections were then incubated overnight with primary goat anti-*CYP11B* Abs (1:300 Santa Cruz) or rabbit anti-*11β-HSD2* Abs (1:300 Biosynthesis Biotechnology) at 4°C. Immunoreactivity was detected using biotinylated secondary antibodies, and the avidin-biotin complex was visualized using DAB (DAB peroxidase substrate kit, Vector Laboratories). Counterstaining was performed with hematoxylin to enhance nuclear detection. Normal goat or rabbit IgG was served as a negative control.

### Enzyme-linked Immunosorbent assay

The concentration of corticosterone was determined using a corticosterone ELISA kit from Cayman Chemical according to the manufacturer’s instructions. The results are expressed in ng/ml of plasma. For placental corticosterone concentrations, frozen placentas stored at − 80°C were thawed, and 100 mg of tissue was homogenized in enzyme immunoassay buffer containing 0.1 M phosphate solution with 0.1% BSA, 0.4 M NaCl and 1 mM EDTA and centrifuged at 1500 g for 15 min at 4°C. The results are expressed as pg/mg of tissue.

### Reverse transcription-PCR analysis of mRNA expression

The placental tissues were homogenized with TRIzol reagent (Invitrogen), and total RNA was extracted according to the manufacturer’s instructions. A reverse transcription-PCR (RT-PCR) procedure was used to determine the relative quantities of mRNA (One Step RT-PCR kit, Qiagen). *GAPDH* was used as the internal control. The primers for all genes tested are listed in Table 1. Amplified cDNA was separated by electrophoresis on a 1.6% agarose gel and identified with Gold View.

**Table 1:** **Oligonucleotides sequences used for RT-PCR**

Target mRNA	Prime	Sequence
*GAPDH*	Forward	5′-GTGGAGATTGTTGCCATCAACG −3′
	Reverse	5′-CAGTGGATGCAGGGATGATGTTCTG −3′
*StAR*	Forward	5′-GAAGGCTGGAAGAAGGAAAG-3′
	Reverse	5′-GAACTCTATCTGGGTCTGTG-3′
*CYP11A1*	Forward	5′- CTGCCTGGGATGTGATTT −3′
	Reverse	5′-GGAAGTGCGTGGTGTTTT −3′
*3β-HSD*	Forward	5′- TCAAATCCATACCCATACAGC-3′
	Reverse	5′- GCCACATTGCCTACATACACT −3′
*CYP21A1*	Forward	5′-ACCTTCCGGTCTACCTGTTT-3′
	Reverse	5′-AAGTGCTGTCCTGCTTGTCT-3′
*CYP11B1*	Forward	5′-CTGCTGGGACATTCGTCA-3′
	Reverse	5′-ACAGGCCGGAAAGTAAGG-3′
*11β-HSD2*	Forward	5′-TCTCCAGTGGTAACTTTCCG-3′
	Reverse	5′-TGCTCAATGTAGTCTTCACC-3′

### Protein isolation and western blot

Protein was extracted from placental tissue using the ProteoJET™ Mammalian Cell Lysis and Cytoplasmic Extraction Kit. Protein concentrations were determined using a BCA protein assay kit. We separated the proteins (50 μg) on an 11.5% gel and transferred them to PVDF membranes (Bio-Rad). Anti-*CYP11B1* (1:500) and *11β-HSD2* (1:500) were applied. Bound antibody was detected by enhanced chemiluminescence on an X-ray film. We analyzed the relative densities of the bands with Image J 1.47. The band densities were normalized to *GAPDH* (glyceraldehyde-3- phosphate dehydrogenase).

### Statistics

All statistical analyses were performed using SPSS 16.0 software. Numerical results are expressed as the mean ± SEM of multiple experiments. We used a mixed-model repeated-measures analysis to compare the characteristics of pregnant rats exposed to different doses of Cd. The means of the different groups were compared by a one-way ANOVA followed by a Tukey multiple comparison test and by the Kruskal-Wallis (when the data were not normally distributed). Differences were considered to be statistically significant when the P value was < 0.05.

## Results

### Characteristics of pregnant mothers

**Figure 1:**
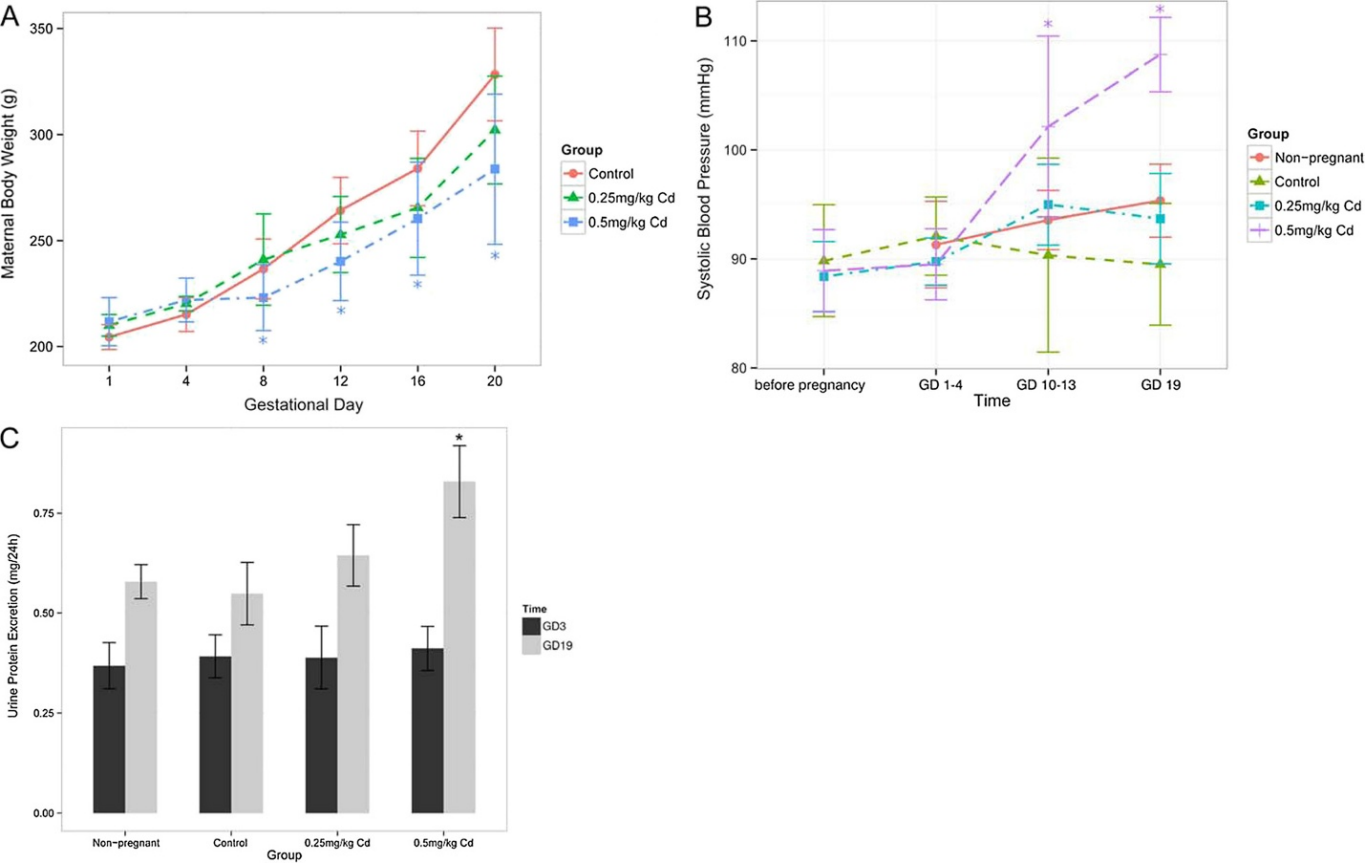
**SD rats develop preeclampsia-like symptoms in response to cadmium.** Pregnant rats were given saline (in control group) or 0.25 or 0.5 mg/kg Cd intraperitoneally on GD 5 to GD 19. Maternal body weight, systolic blood pressure and 24-hour urinary protein excretion are presented in **(A)**, **(B)** and **(C)**, respectively. All data are expressed as the mean ± SEM. *P < 0.05 compared with the control group and non-pregnant rats.

The characteristics of rats subjected to different Cd dose during pregnancy are described in Figure 1. Maternal daily weight gain through the dosing period was significantly lower in the 0.5 mg/kg of Cd treatment group compared to the control group from GD 8 to GD 20. In terms of maternal total weight gain, the group that received 0.5 mg/kg Cd gained significantly less weight compared to the other groups (P <0.05, Figure 1A).

Hypertension is a defining feature of preeclampsia. Figure 1B showed that exposure of pregnant rats to 0.5 mg/kg Cd resulted in a significant pregnancy-dependent increase in systolic blood pressure compared with non-pregnant animals at GD 19 (P <0.01). Systolic blood pressures for pregnant rats exposed to 0.5 mg/kg of Cd were significantly higher than the blood pressures of rats in the control group (P <0.01) and the 0.25 mg/kg of Cd group (P <0.01) at GD 19.

The 24-hr urinary albumin excretion, another defining feature of preeclampsia, was measured in pregnant rats. The results show that a pregnancy-dependent increase in urinary albumin occurred after injection of 0.5 mg/kg Cd in contrast to non-pregnant rats (P <0.01) that received the same treatment, and an increase was not observed in other pregnant groups. Proteinuria in the 0.5 mg/kg Cd group was significantly different from both the control group and the 0.25 mg/kg Cd group (P <0.01, Figure 1C).

### Characteristics of fetuses

We also examined the outcomes from these pregnancies(seen in Table 2). Prenatal Cd exposure did not significantly affect placental weight or implantation size (P <0.05). However, pup weight was significantly reduced in rats from the 0.5 mg/kg Cd group compared with the control group (P <0.05). The 0.5 mg/kg Cd dose resulted in considerably more prenatal losses (due either to resorption and/or stillbirth) (P <0.01). A 28% reduction in fetal body weight and 30% reduction in the litter size of viable fetuses were observed in the 0.5 mg/kg Cd rats compared with control rats (P <0.05). The fetal weight:placental weight ratio and crown rump length in 0.5 mg/kg Cd-treated rats decreased significantly compared with control fetuses (P <0.05; P <0.001, respectively).

**Table 2:** **Effects of cadmium treatment in rats during pregnancy on indices of fetal growth and development**

Group	Placental weight (g)	Implantation size (n)	Resorbed and stillborn fetuses	Number of live fetuses	Fetal weight (g)	Fetoplacental index (g/g)	Crown-rump length (cm)
Control	0.51 ± 0.09	12.5 ± 2.3	0.17 ± 0.41	12.3 ± 2.6	3.53 ± 0.67	7.00 ± 0.94	3.62 ± 0.1
Cd^2+^0.25	0.48 ± 0.06	11.6 ± 1.4	0.57 ± 0.79	11.0 ± 1.9	3.14 ± 0.56	6.56 ± 0.83	3.17 ± 0.35*
Cd^2+^0.5	0.46 ± 0.05	10.9 ± 1.8	2.25 ± 2.05*	8.6 ± 3.5	2.51 ± 0.61*	5.52 ± 1.33*	2.74 ± 0.36**

Data are the mean ± SEM (n = 6–8 pregnant mice).

*P < 0.05 compared with the control group.

**P < 0.01 compared with the control group (one-way ANOVA followed by a Tukey multiple comparison test or by the Kruskal-Wallis when the data were not normally distributed).

### Morphological changes in the placenta , decidua and kidney with preeclampsia

**Figure 2:**
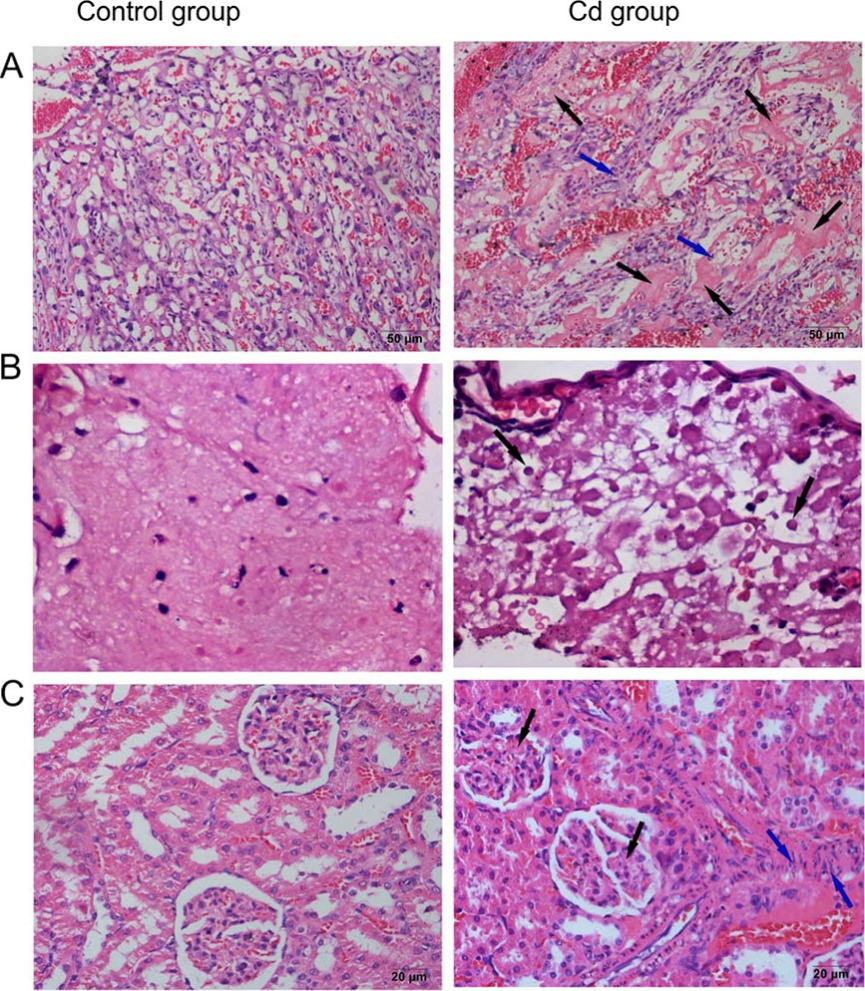
**Hematoxylin-eosin stain for placental, decidua and kidney pathological analysis.** Placenta, decidua and kidney specimens were obtained from normal pregnancy rats and 0.5 mg/kg Cd-treated rats on day 20 of pregnancy. Left side photos are from the control group and right side photos are from Cd-treated group. Hematoxylin-eosin staining was used for pathological analysis. **(A)** Representative images of the placenta (original magnification X200). Black arrows indicated degeneration and excessive perivillous fibrin deposition in the placental labyrinth. Blue arrows indicated leukocytes. **(B)** Representative images of the decidua (original magnification X400). Black arrows indicated vacuolization and swelling decidua. **(C)** Representative images of the kidney (original magnification X400). Black arrows indicated glomerular endotheliosis. Blue arrows indicated thickening of the media of renal vessel walls.

As showed in Figure 2, Cd treatment caused placental thickening in the media of vessel walls and degeneration and excessive perivillous fibrin deposition in the placental labyrinth. Additionally, vacuolization and swelling decidua were found in the 0.5 mg/kg Cd-treated group but were absent in the control group.

Kidneys from the 0.5 mg/kg Cd-treated group displayed swelling of endothelia cells that appeared to reduce capillary space (glomerular endotheliosis) and thickening of the media of renal vessel walls. Typical changes in the maternal endothelium due to preeclampsia induced by Cd were observed in the pregnant rat kidney. Accumulation of inflammatory leukocytes was both observed in the placenta and kidneys.

### Cadmium concentrations

As expected, Cd levels in the placenta were significantly higher for the 0.5 mg/kg Cd-treated group (2.81 ± 0.5 μg/g dry weight) than the 0.25 mg/kg Cd-treated group (1.03 ± 0.3 μg/g dry weight, P <0.05). Cd was also detected in the blood of mothers (30.6 ± 0.2 μg/L) in 0.5 mg/Kg Cd-treated group. The Cd in the blood of fetuses whose mothers were exposed to Cd, as well as in the blood of mother in the control group was under the detectable level.

### Circulating and local corticosterone concentrations

Because corticosterone is the main glucocorticoid in rats, we determined corticosterone concentrations in plasma of mothers and fetuses at GD 20 and also in placentas collected after caesarean birth. Plasma corticosterone concentrations were significantly increased after prenatal exposure to 0.5 mg/kg Cd compared to the control group in both pregnant rats and their offspring (P <0.01, Table 3). The concentration of corticosterone in the local placental tissue was also increased in the 0.5 mg/kg Cd injection group (P <0.01). These data indicate that PE-like features and intrauterine growth restriction (IUGR) were present in the groups with high circulating and local corticosterone levels. The minimum level of detection of the assay was 150 pg/ml.

**Table 3:** **Effect of cadmium treatment in rats during pregnancy on placental corticosterone concentration**

Group	Maternal plasma (ng/ml)	Offspring’s plasma (ng/ml)	Placenta (pg/mg tissue)
control	142.1 ± 15.5	115.9 ± 5.1	79.7 ± 5.2
Cd^2+^0.5	231.1 ± 12.9*	168.9 ± 9.4*	143.4 ± 11.0*

Results are expressed as the mean ± S.D. Significant differences between groups, Wilcoxon rank sum test for independent samples. *P < 0.01 compared with the control group.

### Corticosterone biosynthesis pathway

**Figure 3:**
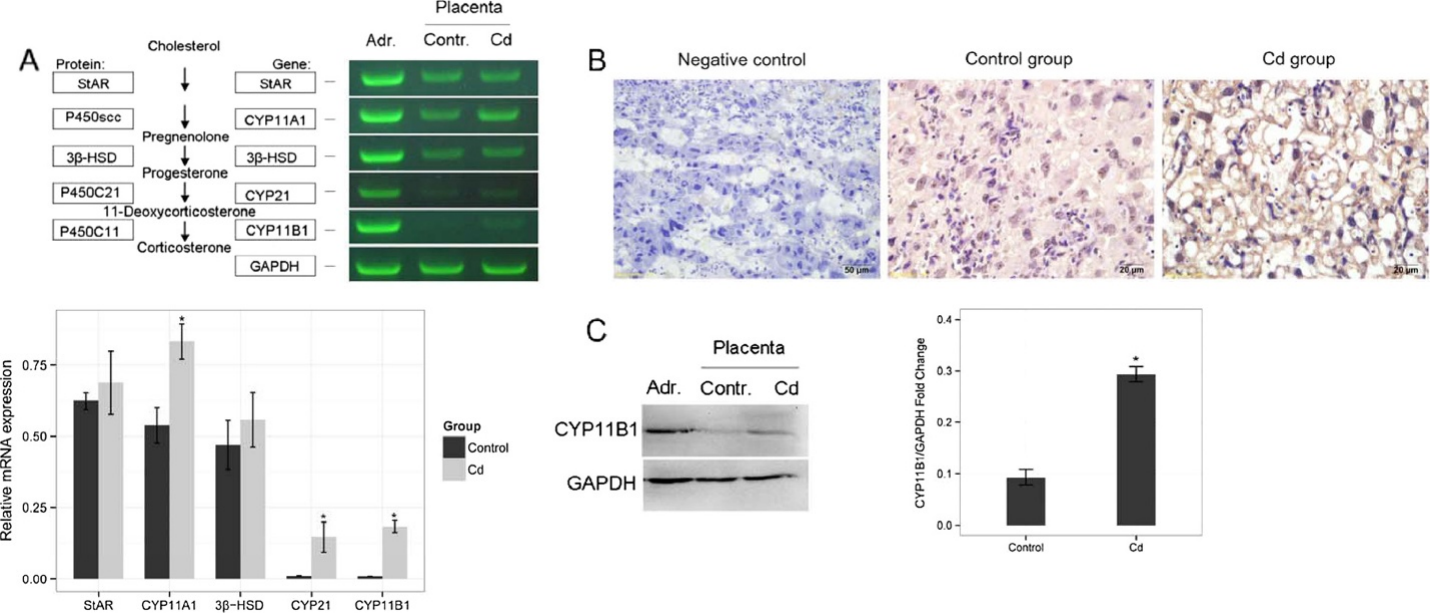
**Expression of steroidogenic enzymes in placental tissue.** The expression of steroidogenic enzymes in placental tissue was detected in the 0.5 mg/kg Cd treatment group. The representative photographs for PCR or Western bolt were from one of three independent experiments that yielded similar results. *GAPDH* was used as the loading control. **(A)** Simplified scheme of the rat glucocorticoids biosynthesis pathway. Detection of steroidogenic enzyme gene expression by RT-PCR in rat adrenal gland or placenta. Comparison of the expression of steroidogenic enzymes in the rat placenta. *P < 0.05 compared with the control group. **(B)** Immunohistochemical localization and expression of *CYP11B* enzymes in the labyrinth zone of rat placenta (original magnification X400). **(C)** Comparison of the expression of *CYP11B1* protein levels in the rat placenta based on Western blot. *P < 0.01 compared with the control group.

As outlined in Figure 3A, we observed that *CYP11A1* was slightly increased in the 0.5 mg/kg Cd treatment group (P <0.05). The levels of *CYP21* and *CYP11B1* mRNA expression in normal placenta tissue were below detectable levels. In contrast, a low level of *CYP21* and *CYP11B1* mRNA was observed in the placenta of the 0.5 mg/kg Cd treatment group (P <0.01). To investigate whether the increased mRNA expression also resulted in increased protein expression, we determined *CYP11B1* protein expression by immunohistochemical staining and Western blot. Our study revealed low levels and sparsely distributed *CYP11B* primarily localized in placental labyrinthine trophoblasts. Weak but significantly higher *CYP11B1* protein expression was detected in the placenta of the 0.5 mg/kg Cd treatment group (P <0.01). We also compared the amount of mRNA of each steroidogenic enzyme in the rat placenta with that in the rat adrenal gland. The concentrations of *CYP21* and *CYP11B1* in the placenta were much lower than those in the adrenal gland (data not shown).

The direct demonstration of the presence of the whole set of enzymes necessary for glucocorticoids production in placenta supports the ability of the rat placenta to synthesize the local production of corticosteroids after exposure to Cd.

### Regulation of glucocorticoid activity by 11β-HSD in the placenta

**Figure 4:**
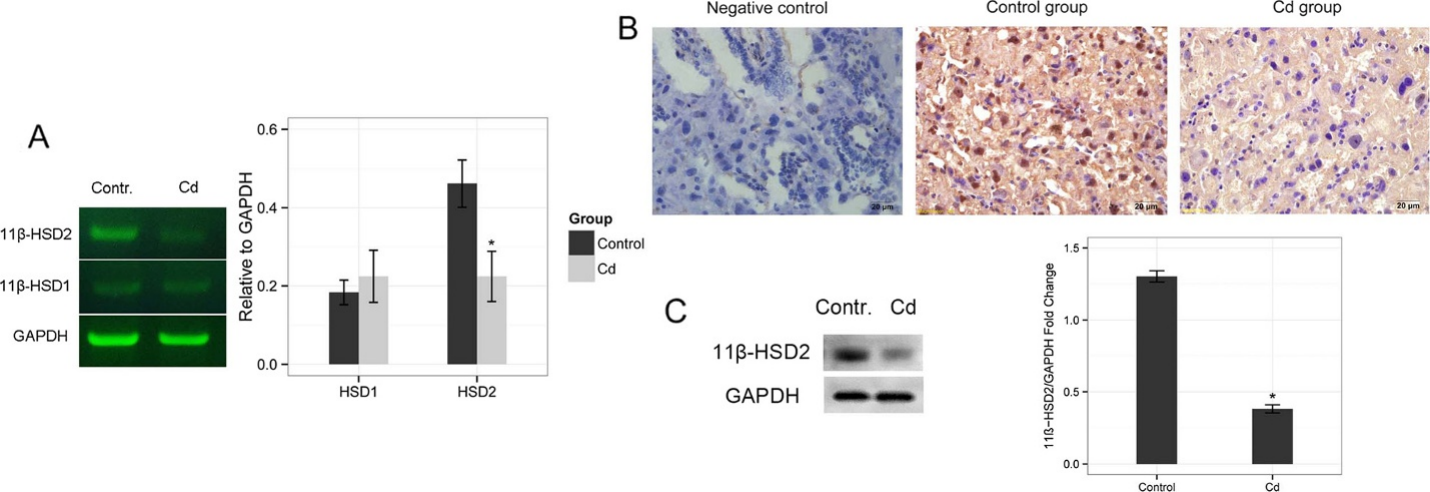
**Regulation of glucocorticoid activity by 11β-HSD in the placenta.** Downregulation of *11β-HSD2* (negative feedback enzyme) is induced by Cd in the rat placenta. The representative photographs for PCR or Western blots were from one of three independent experiments that yielded similar results. *GAPDH* was used as the loading control. **(A)** *11β-HSD1* and *11β-HSD2* mRNA levels were analyzed by RT-PCR in the rat placenta. The expression of *11β-HSD1* and *11β-HSD2* mRNA was compared between the control group and the 0.5 mg/kg Cd treatment group. *P < 0.01 compared with the control group. **(B)** Immunohistochemical staining of *11β-HSD2* protein in the labyrinth zone of placenta (original magnification ×400). **(C)** The expression of *11β-HSD2* was analyzed by Western blot in the rat placenta. *P < 0.01 compared with the control group.

11-hydroxysteroid dehydrogenase (*11β-HSD*) is a dominant regulator of local glucocorticoid levels. *11β-HSD1* generates active glucocorticoid through catalyzing the conversion of 11-dehydrocorticosterone to corticosterone and cortisone to cortisol. In contrast, placental *11β-HSD2* protects the fetus from the adverse effects of maternal glucocorticoids by interconverting the active glucocorticoids to their inactive metabolites. Surprisingly, when compared with the control groups, the expression of *11β-HSD1* was not changed (P =0.14), whereas *11β-HSD2* was significantly reduced in the placenta of Cd-treated rat at both the mRNA and protein levels (P <0.01, Figure 4). *11β-HSD2* was mainly localized in the cytoplasm of the labyrinthine and spongiotrophoblasts layers of the placenta. The above data suggest that prenatal exposed to Cd may induce the downregulation of *11β-HSD2*, leading to increased local glucocorticoid activity in the placenta.

## Discussion

Previous studies have shown that hypertension was induced by the intraperitoneal administration of 1 mg/kg/day Cd for 15 days in non-pregnant rats [18, 19]. Hypothesizing that pregnant rats are more susceptible to Cd toxicity, we exposed pregnant rats to relatively low dosages of Cd and evaluated them for maternal-placental-fetal parameters linked to preeclampsia. We demonstrated that the hallmarks of preeclampsia, including acute maternal hypertension, hyperalbuminuria and IUGR, were found in SD rats exposed to 0.5 mg/kg Cd during gestation (GD5-19). The typical changes in the maternal endothelium in preeclampsia ere also observed in the rat kidney after Cd treatment. The reduction in fetal weight independent of placental weight in the 0.5 mg/kg Cd-treated group has been postulated to result from an abnormal placenta with impaired function and reduced perfusion after fetal and/or placental exposure to Cd.

To explore the biochemistry underlying these physiological changes, we used a very sensitive ELISA to detect placental corticosterone. A marked increase in the concentration of placental corticosterone was observed in rats treated with Cd. High corticosteroid concentrations in extra-adrenal organs, sometimes much higher than circulating concentrations, is evidence of local synthesis. Despite the presence of *11-HSD*, which is involved in the upregulation of glucocorticoids in the placenta [20], in our opinion it is also possible that a classical glucocorticoid biosynthesis pathway may be involved in these processes. To date, few studies have examined the levels of steroidogenic enzymes necessary for *de novo* synthesis of corticosterone from cholesterol in the placenta [21].

The placenta utilizes maternal cholesterol as the initial substrate to produce progesterone for the duration of the pregnancy, and most of the produced progesterone enters the maternal circulation [22]. Progesterone is also a source of corticosteroid precursors. *StAR* is a protein involved in the shuttling of cholesterol to the inner mitochondrial membrane for enzymatic conversion. In the placenta, cytochrome *P450scc* (*CYP11A1*) is the most important and rate-limiting enzyme for the conversion of cholesterol to pregnenolone [23]. 21-hydroxylase (*CYP21*) and 11-beta-hydroxylase (*CYP11B1*) are required for corticosterone synthesis; they convert progesterone to corticosterone. According to our research, although there was a slight increase in *CYP11A1* mRNA, there was no significant change in *StAR* and *3β-HSD* mRNA after exposure to Cd, indicating that 0.5 mg/kg Cd does not affect placental gonadal steroidogenesis, especially progesterone biosynthesis, in rats. *CYP11B1* is required for the final steps of glucocorticoids synthesis and is followed by the activity of the *CYP21* enzyme [24]. In our studies, the expression of *CYP21A* and *CYP11B1* mRNA in normal rat placenta tissue was too low to be detected consistently and reproducibly, so the ability of normal placenta to produce corticosterone is very limited, which is in agreement with the data reported by Sybulski [25]. After Cd injection, *CYP21A* and *CYP11B1* mRNAs in the placenta increased, although the levels were still lower levels than those in the adrenal gland. However, it should be remembered that because the placenta is much larger than the adrenal gland, the total mRNA for glucocorticoid-synthesizing enzymes in the entire placenta could not be underestimated. The enzyme *CYP11B1* was detected rather easily using immunohistochemistry or western blot, suggesting that the protein is stable in the placenta. The expression of either of these enzymes would enable the placenta to use circulating steroid precursors or local progesterone to increase the local levels of active glucocorticoids, which influence placental structure and function. The mechanisms by which Cd affect gene expression may include mimicry of the effects of Ca^2+^ at low concentrations of Cd^2+^ due to the similarity of the ionic radii [26], resulting in stimulation of the steroidogenic pathway and synthesis of corticosteroids. We proved that local corticosteroid synthesis may be activated under pathological conditions in placenta.

In normotensive pregnancy, glucocorticoids are inactivated but are frequently present after downregulation of *11β-HSD2* by adverse prenatal experiences [27, 28]. We found that the placenta of rats with preeclampsia during pregnancy is characterized by decreased *11β-HSD2* with unchanged *11β-HSD1*. Cd can directly inhibit the transcription of the *11β-HSD2* gene [29]. This local increase in the utility of glucocorticoids is very important because it allows increased ability of active glucocorticoids to cross the placenta barrier, which accounts for the elevated fetal corticosterone concentrations associated with low birth weight.

In conclusion, this study demonstrates for the first time that the placenta may be an extra-adrenal site of glucocorticoid synthesis due to prenatal Cd exposure in rats. Most of the reviewed research findings suggest that in micromolar concentrations Cd causes upregulation of the mediators and markers of inflammation and appears to have proinflammatory properties [30]. Increasing the local concentration of glucocorticoids is a protective mechanism to control the deleterious effects of excessive production of inflammatory mediators. Although we feel that the present results are encouraging, further studies are needed to elucidate the clinical implications of extra-adrenal corticosteroidogenesis in the preeclamptic placentas of Cd-exposed pregnant rats.

## Conclusions

We found that prenatal exposure to a relatively low concentration cadmium will develop many of the phenotypic characteristics of human preeclampsia, including hypertension, proteinuria,and fetal growth restriction (FGR) in rat. We present the first evidence that cadmium exposure during pregnancy induces local synthesized glucocorticoid in placenta and contributes to preeclampsia in rat. This is a potentially important hypothesis for experimental investigation, given the relevance of human exposure to Cd and its hazard to pregnancy.
